# Global implication of mesoproterozoic (~ 1.4 Ga) magmatism within the Sette-Daban Range (Southeast Siberia)

**DOI:** 10.1038/s41598-021-00010-5

**Published:** 2021-10-14

**Authors:** Sergey V. Malyshev, Alexei V. Ivanov, Andrey K. Khudoley, Alexander E. Marfin, Vadim S. Kamenetsky, Maya B. Kamenetsky, Olga Yu. Lebedeva

**Affiliations:** 1 Institute of Earth Sciences, Saint Petersburg University, University Emb. 7-9, Saint-Petersburg, Russia 199034; 2 Institute of the Earth’s Crust, Siberian Branch of the Russian Academy of Sciences, Lermontova St. 128, Irkutsk, Russia 664033; 3 School of Natural Sciences and Centre for Ore Deposit and Earth Sciences (CODES), University of Tasmania, Hobart, TAS 7001 Australia; 4 Institute of Experimental Mineralogy, Russian Academy of Sciences, Akademika Osip’yana st. 4, Chernogolovka, Russia 142432; 5 A.P. Karpinsky Russian Geological Research Institute, Sredniy pr., 74, Saint-Petersburg, Russia 199106

**Keywords:** Precambrian geology, Geochemistry, Tectonics

## Abstract

Mesoproterozoic period included several global tectonic events like break-up of Nuna and formation of Rodinia. However, although Siberia is a significant piece of both supercontinents, Mesoproterozoic time is marked by quiescence of magmatic and tectonic activity in it. We report here a mafic dyke (named Gornostakh dyke) in the southeastern Siberian Craton dated at 1419 ± 32 Ma by LA-ICPMS U–Pb geochronology of apatite. The dyke has tholeiitic compositions with high MgO and alkaline content, low-Ti, and arc-like trace element pattern. Due to the absence of subduction tectonics in the study area, geochemical data could be attributed to a significant contribution from metasomatically enriched subcontinental lithospheric mantle previously modified by subduction processes. That kind of composition is common for low-Ti dykes of intraplate flood basalt provinces similar to, for example, Permian–Triassic Siberian large igneous province (LIP). Paleogeographic reconstructions suggest that Siberia was connected to Laurentia and Baltica and their reconfiguration interrupts a prolonged tectonic quiescence in the Siberian Craton from ca. 1.88 Ga reflecting a transition from Nuna to Rodinia configuration. The mafic magmatism on 1419 Ma on the southeastern margin of the Siberian Craton together with coeval extensional tectonics observed in the structure of the Sette-Daban ridge proposes a hypothetical LIP which may be a direct consequence of the beginning of this transition.

## Introduction

Mesoproterozoic is included in the time period which is called the Boring Billion (1.70–0.75 Ga)^1,2^, which was marked by an absence of glacial deposits and Sr anomaly in the seawater record, lack of massive sulfide volcanic-hosted deposits and iron formations, lack of orogenic gold^3^. The paucity of new passive continental margins and abundance of unusual dry magmas such as A-type granites and anorthosites in Mesoproterozoic are discussed in terms of ‘lid tectonic’ hypothesis^4^. According to the paleomagnetic reconstructions in Mesoproterozoic, all of the continents were assembled into the supercontinent Nuna (Columbia) which were reassembled to Rodinia at the end of Mesoproterozoic e.g.^5–9^, and reference within. Large Igneous Provinces (LIPs) and regional mafic dyke swarms as a component of LIPs act as a significant fingerprint for supercontinental reconstructions and their presence indicates tectonic markers of intracratonic crustal extension associated with deep-Earth dynamic processes such as mantle plumes, subduction (back-arc extension), and rifting during supercontinent break-up^10–13^.

Outcrops of Paleoproterozoic or early Mesoproterozoic crust in Siberia are restricted to isolated exposures of basement inliers and surrounding areas or lower Mesoproterosoic sediment cover of foreland Verkhoyansk fold-and-thrust belt (FTB). The largest dyke swarms or extensive sills are located within the basement inlier in Anabar uplift and Olenek uplift and their surrounding areas, and Sette-Daban range of Verkhoyansk FTB (with ages ~1500 Ma and 1000–950 Ma, respectively^14,15^). The period of 1400–1300 Ma is marked by a few mafic intrusions across the Siberian craton: 1382 ± 2 Ma Chieress dyke cut the Mesoproterozoic sediment cover of the eastern slope of Anabar uplift^16^, 1385 ± 30 Ma mafic dykes cut the Mesoproterozoic sediments on the Udzha uplift^17^, Listvyanka and Goloustnaya dykes exposures in the south margin of the Siberian craton – 1350 ± 6 Ma and 1338 ± 3 Ma, respectively^18^ and reference within.

Another poorly dated north-trending dolerite dyke at south Verkhoyansk FTB was explored. This dyke cuts the lower Mesoproterozoic sediments (Lower Riphean according to the Russian stratigraphic nomenclature). The age of this dyke was estimated as ca. 1.34 Ga using Sm-Nd isochron^19^. We report here a new U-Pb age on apatite (1420 Ma), Nd-isotopes, and major and trace element geochemistry for this dolerite dyke to constrain mantle source and a possible link of this magmatic event with continental reconstruction in Mesoproterozoic.

## Regional geology and sample description

**Figure 1:**
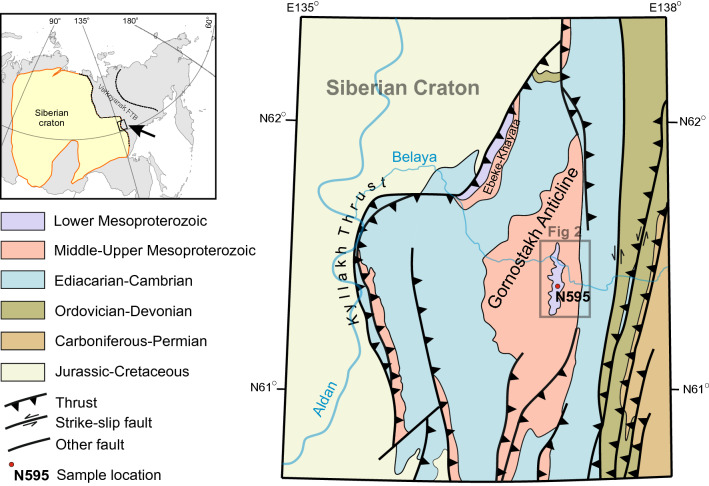
Simplified geological and sample location map of the central part of Sette-Daban Range and surrounding areas (after^20,25^, simplified and modified).

The eastern part of the Siberian craton is separated from the Verkhoyansk FTB by a set of thrusts. The southernmost part of the Verkhoyansk FTB is widely known as the South Verkhoyansk segment^20^. The study area is located in the western part of this segment, within the Sette-Daban Range (Fig. 1).

Within the Sette-Daban area, the basement is totally covered by Mesoproterozoic to Jurassic sedimentary succession. Meso- and Neoproterozoic rocks are exposed in the hanging walls of major thrusts which specifies the predominant structural style as imbricate thrust sheets. From the south to the north several thrusts pinch out, forming the wide Gornostakh Anticline with a gentle (10°–15° dip angle) west limb and a much steeper (40°–50° dip angle) east limb (Fig. 1).

The Meso- and Neoproterozoic succession contains several kilometer-scale distinctive siliciclastic-carbonate cycles that are generally separated from each other by unconformities or erosional surfaces and are divided into the following six widely recognized units: the Uchur, Aimchan, Kerpyl, Lakhanda, Uy and Yudoma Groups^21^. A detailed description of the sedimentary succession of the Sette-Daban region has been presented in several papers, but the important thing for this study is that only Uchur Group is estimated as lower Mesoproterozoic in age, whereas others have middle Mesoproterozoic to Ediacarian ages^22^ and reference within. The outcrops of the lower Mesoproterozoic succession (Uchur Group) are limited to isolated exposures within the Verkhoyansk FTB and represented by two locations: in the core of the Gornostakh Anticline and in the hanging wall of the northern branch of the Kyllakh Thrust (Ebeke-Khayata Range, see on Fig. 1). The only angular unconformity (with angle ~20°) in the Mesoproterozoic sequence is observed between the Uchur and Aimchan Groups, and it reflects the only Mesoproterozoic extension deformation event in the region^21^.

**Figure 2:**
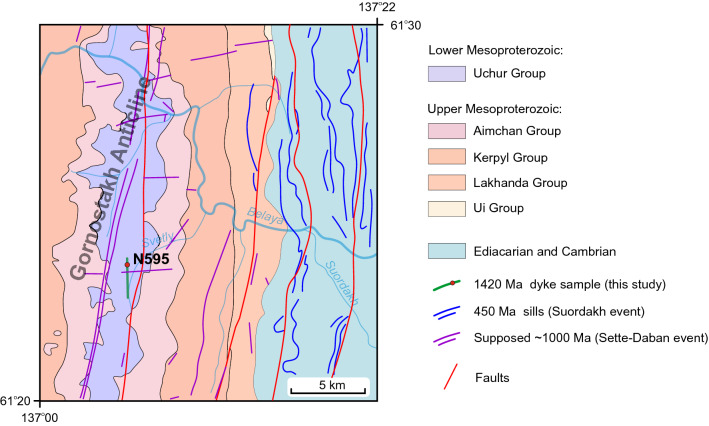
Geological setting of the central part and eastern limb of the Gornostakh Anticline (Belaya River area) and location of sampled dolerite dyke in this study (geology after^25^, modified and simplified). Ca 450 Ma sills (Suordakh event) from^24^.

Three major mafic magmatic events are recognized in the Sette-Daban: 1000–950 Ma Sette-Daban event, 440–450 Ma Suordakh event and 370–360 Ma Yakutsk-Vilyui LIP^15,23,24^ (Fig. 2). Gornostakh dyke analyzed in this study according to the geological maps crosscuts only the sediments of the Uchur Group in the core of the Gornostakh Anticline. A preliminary Sm-Nd isochron age of the Gornostakh dyke with the relatively large analytical error is 1339 ± 59 Ma^19^. If correct, this age suggests another mafic magmatic event in the area and the lack of exposure of the lower Mesoproterozoic sedimentary rocks in the Sette-Daban allows considering the Gornostakh dyke as a unique intrusion for assessing the nature of the lower Mesoproterozoic magmatism in the region.

**Figure 3:**
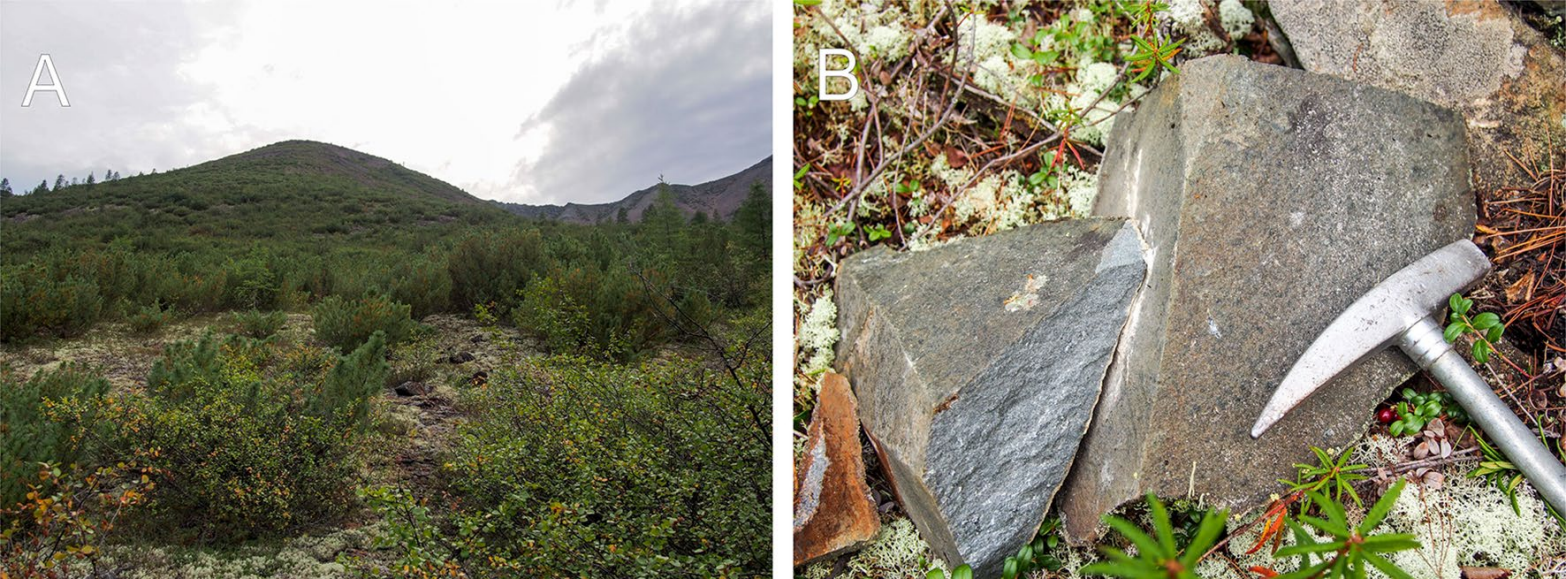
(**A**) A view of the sample location, looking NNW. (**B**) Dolerite sample N595.

The field sampling site was targeted using 1:200 000 geological maps of the USSR, sheet P-53-XXIV^25^. The dolerite sample was collected from the western slope of the Svetly Creek valley, approximately 7 km from its confluence with the Belaya River (Fig. 2). Dyke has no bedrock exposure but does have concentrations of large boulders of dolerite forming a north-trending line, which is shown on maps as a dyke. Due to the lack of exposed contacts, the width of the dyke are unknown, but estimated roughly as 15-20 meters. All rocks are relatively fresh, and weathering forms a thin pale brown crust which is developed along with fractures (Fig. 3).

**Figure 4:**
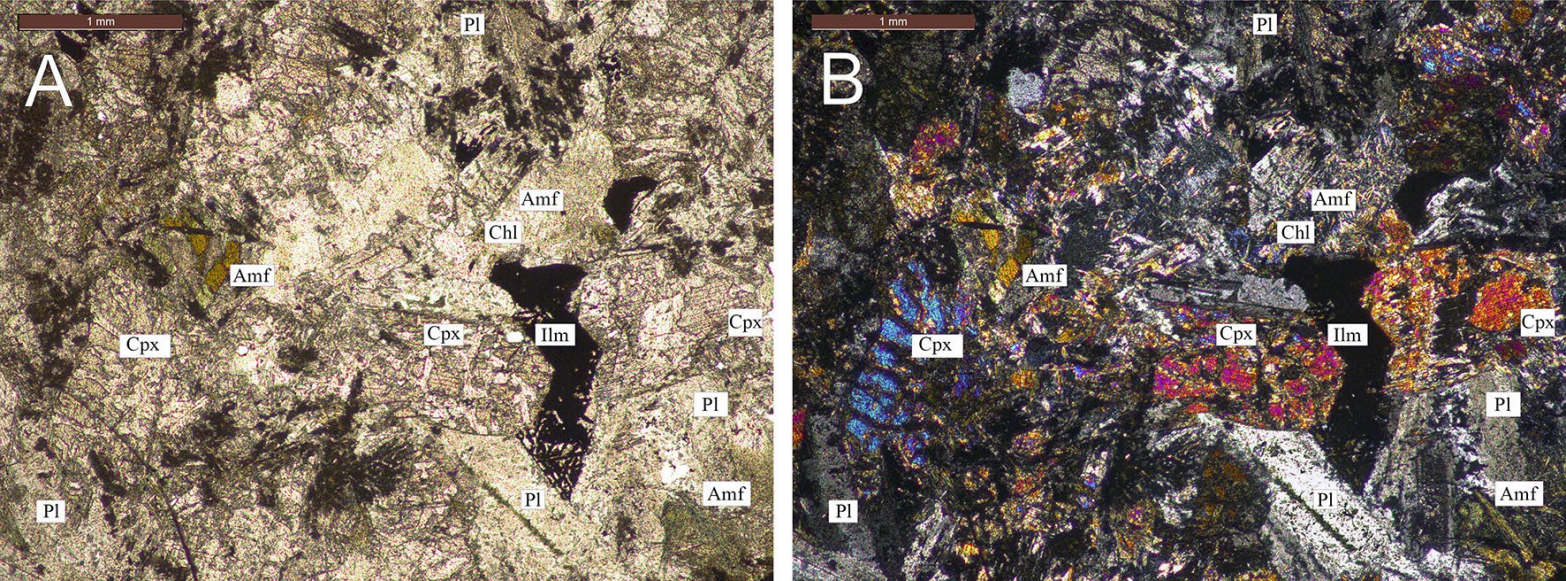
Photomicrograph of sample N595 (Gornostakh dyke). (**A**) Transmitted polarized light. (**B**) Cross-polarized light. Pl = plagioclase, Cpx = clinopyroxene, Amf = amphibole, Chl = chorite, Ilm = ilmenite.

The sample is dolerite with intergranular ophitic to sub-ophitic texture, comprising 50–55% plagioclase, 40–45% clinopyroxene, up to 0.5% quartz and 4–5% opaque minerals (ilmenite) and trace apatite grains (Fig. 4). The sample is moderately altered with uralitic alteration of pyroxene and variable moderate sericitization of plagioclase. Most of clinopyroxene grains have been affected by alteration (80% of the grain volume) and are present in the form of relics broken by cracks or pseudomorphisms, completely overgrown by chlorite and amphibole. Amphibole grows from near grain boundaries to pervasive alteration of the entire grain into a mixture of colorless actinolite-tremolite and greenish-brown xenomorphic grains of hornblende. Plagioclase (andesine №35–37) preserves original polysynthetic twinning and the entire volume of grains is permeated with a thin aggregate of saussurite and thin needles of actinolite-tremolite. Opaque minerals appear as subhedral to euhedral grains of ilmenite variable in size (0.1–2 mm) in groundmass and also as extremely fine-grained masses within altered pyroxene and along grain boundaries. Accessory apatite grains form thin prisms and needles up to 0.5 mm in size.

## U–Pb geochronology and geochemistry methodologies

Heavy mineral fractions were extracted at the Centre of Geodynamics and Geochronology at the Institute of the Earth's Crust Siberian Branch of the Russian Academy of Sciences. Therefore patite grains were employed for U-Pb dating, which was performed at the University of Tasmania (Hobart, Australia) by laser ablation inductively coupled plasma mass spectrometry (LA-ICPMS) using an Agilent 7900 quadrupole mass-spectrometer connected to a 193 nm Coherent Ar-F excimer laser and Resonetics S155 ablation cell^26,27^. Details of the procedure are provided in the Supplementary File along with the full analytical dataset.

The isotopic study was done at the Institute of Geology and Geochronology of the Precambrian, Russian Academy of Sciences. Accuracy at 2σ level of ^147^Sm/^144^Nd and ^143^Nd/^144^Nd ratio measurements was 0.5% and 0.005%, correspondently. ^143^Nd/^144^Nd ratios were reported relative to the value of 0.511860 for the La Jolla standard. During the period of this study, the weighted average of nine La Jolla Nd-standard runs yielded 0.511852±8 (2σ, n=10) for the ^143^Nd/^144^Nd ratio after normalization of the ^146^Nd/^144^Nd ratio to 0.7219.

## Results

### LA-ICPMS U–Pb geochronology

**Figure 5:**
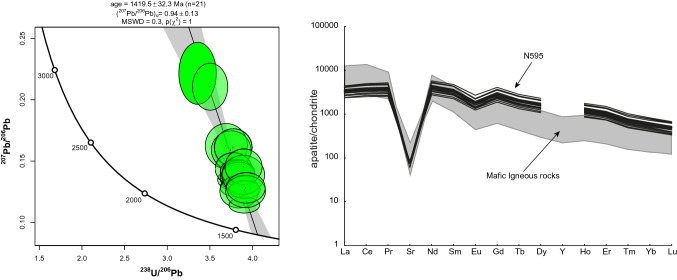
Tera-Wasserburg concordia diagram for the dated apatite grains (on left). Weighted mean age is presented with 1 s error. In discussion we used 2 s error. Chondrite normalized rare earth elements spectra for analyzed apatite grains (on right). Grey field denotes to a typical range of apatite REE compositions from mafic host rocks^29^. Raw data presented in supplementary file.

A typical apatite grains were used for the U-Pb dating. The apatite grain was large enough to employ 30 µm diameter laser ablation spots to grain regions free of mineral and melt inclusions. On the Tera-Wasserburg diagram, the analyzed apatite grains are discordant and form a pronounced regression line that intercepts in the lower part with the concordia at 1419 ± 32 Ma (Fig. 5). The trace element composition of apatite can be used to clarify the initial composition of magma, which has been shown in numerous studies^28,29^ and reference within. The multi-elemental spectra in the studied apatite indicate the closeness of the trace element composition of the grains. A characteristic feature of those apatite grains is the presence of pronounced Sr and Eu negative anomalies, which are typical for apatite from mafic igneous rocks.

### Geochemistry

Major and trace element data for the studied sample N595 are presented together with temporally closest 1385 Ma Udzha dyke from northern Siberia and 1340 Ma Listvyanka dykes from Irkutsk Promontory^17,18^.

**Figure 6:**
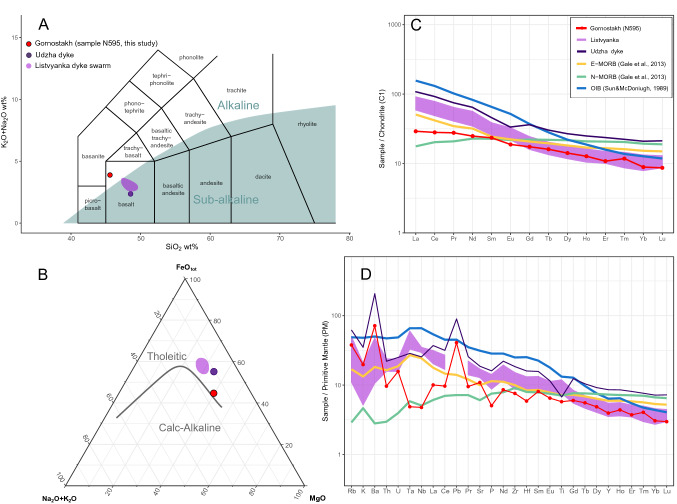
(**A**) Total alkali-silica (TAS) plot after^31^ with alkaline-sub-alkaline boundary after^30^. Purple field the ca. 1340 Ma from Listvanka dykes from^18^, 1385 Ma Udzha dyke from^58^. (**B**) AFM plot after^30^. (**C**) Chondrite and (**D**) primitive mantle normalised multi-element plots for Gornostakh (N595), Udzha dyke^58^ and Listvyanka dykes^18^. OIB = ocean island basalt after^46^ NMORB = mid ocean ridge basalt and EMORB = enriched MORB after^59^.

The sample of Gornostakh dolerite display relatively high MgO (9.83 wt%) and Al_2_O_3_ (15.4 wt%), moderate SiO_2_ (45.6 wt%), FeOt (11.1 wt%) and CaO (7.86 wt%), and low-Ti content (1.25 wt%) (Table 1). The sample has moderate total alkalis (Na_2_O + K_2_O = 3.9 wt%) and Na_2_O/K_2_O ratio of 2.27. The sampled dyke is classified as alkaline basalt on the TAS diagram (Fig. 6A^30,31^) and plots transitional between tholeiitic and calc-alkaline series on the AFM diagram (Fig. 6B^30^). The chondrite-normalized rare earth element pattern (Fig. 6C) shows moderate enrichment of light REE (LREE) with La_N_/Yb_N_=3.28 where LREE profile is flat with La_N_/Sm_N_=1.24 slightly higher than the average values of N-MORB and lower than average values of E-MORB, whereas the heavy REE (HREE) profiles shows gentle slope with Tb_N_/Yb_N_=1.81. The primitive mantle-normalized trace element patterns (Fig. 6D) show depletion of high field strength elements (HFSE) with prominent negative Nb-Ta and slightly negative Zr-Hf and Ti anomalies and enrichment in Pb, Rb and Ba.

**Table 1:** Major, trace element and isotope data for the sample N595.

XRF	N595 (wt %)	ICP-MS	N595 (ppm)	ID-TIMS	N595
SiO_2_	45.6	Rb	24	^147^Sm/^144^Nd	0.1709
TiO_2_	1.25	Sr	230	^143^Nd/^144^Nd	0.512391
Al_2_O_3_	15.4	Y	18	ε_Nd_(0)	− 4.8
Fe_2_O_3_	2.82	Zr	85	ε_Nd_(t)	− 0.1
FeO	8.54	Nb	3.4	T(Nd)_dm_ (Ma)	2697.6
MnO	0.15	Ba	500		
MgO	9.83	La	6.9		
CaO	7.86	Ce	17.17		
Na_2_O	2.7	Pr	2.63		
K_2_O	1.19	Nd	11.59		
P_2_O_5_	0.11	Sm	3.59		
LOI	4.28	Eu	1.09		
Total	99.7	Gd	3.58		
		Tb	0.6		
FeO_(total)_	11.07	Dy	3.59		
Mg#	61.3	Ho	0.72		
		Er	1.79		
		Tm	0.3		
		Yb	1.51		
		Lu	0.22		
		Hf	1.82		
		Ta	0.2		
		Pb	2.9		
		Th	0.82		
		U	0.33

The Gonstostakh dyke sample was analyzed for Nd isotopes. The corresponding initial εNd_1420Ma_ value shows a slightly negative value ($-$ 0.1), which is much lower than the inferred for the depleted mantle, suggesting involvement of an enriched reservoir (crustal component or enriched subcontinent lithospheric mantle).

## Discussion

The age of mafic (or alkaline ultramafic) rocks is a key for supercontinent reconstructions and global correlation of ancient mafic magmatic activity worldwide^32,33^. Within Siberia mafic magmatism within an uncertainty of 1420 ± 30 Ma age for the Gornostakh dyke is currently unknown in the Sette-Daban or elsewhere in Siberia. The temporally closest magmatic events within the Siberia are the ca 1384–1386 Ma Chieress and Udzha dyke in its northern part^16,17^, the ca. 1350 Ma Listvyanka and Goloustnaya dykes^18^ and reference within in its south part and the 1490–1500 Ma Kuonamka LIP within north Siberia^14^. On a global scale, the 1.42 Ga Gornostakh dolerites may be compared directly with contemporaneous components in different blocks, including Baltica^34^, Laurentia^32,35–37^, Congo^38,39^ and West African^40^ cratons, and craton Yilgarn^41^.

### Mantle sources

Evaluation of mobility of major and trace elements during alteration or metamorphism can be assessed using Zr, Nb, and Y, e.g.^42,43^. Despite moderate growth of secondary minerals in dolerite of the dyke, the Zr/TiO and Nb/Y ratios (68 and 0.19) correspond to subalkaline basalts according to classification by^42^ indicating that immobile trace elements (Nb, Y, Zr, Ta, Th, REE) were unaffected by post-magmatic processes and reflect the primary composition of magma. The Gornostakh dyke displays arc-like geochemical characteristics, including depletion of HFSE, enrichment of LILE, and unradiogenic initial Nd isotopes, which may have been conferred either by crustal contamination or derived from the metasomatically enriched source, or both^44,45^. Significant crustal contamination appears unlikely, which is supported by primitive mantle-like trace element ratios of Nb/Ta (17.0) and Zr/Sm (23.7) ratios (primitive mantle: Nb/Ta=17.39, and Zr/Sm=25.23^46^). This is supported further by La/Sm (1.9) and Sm/Nd (0.31) ratios, and relatively high Mg# (61.3), while crustal material is characterized by high La/Sm and low Sm/Nd relative to rocks sourced from the asthenospheric mantle. Nonetheless the dyke displays arc-like trace element signatures such as the depletion of HFSE and enrichment of LILE, and low Ti. However, subduction also appears unlikely according to paleotectonic reconstructions, which displays intra-plate tectonic setting of the region and absence of active margins around the Siberia. These characteristics may also be attributed to Earth deep volatile cycling^47^ or partial melting of SCLM (sub-continental lithospheric mantle) enriched by ancient recycled components due to subduction processes^44,45^ or low continental crust delamination^48^. Based on the above geochemical and paleotectonic evidence, we prefer an interpretation where the predominant source of the dyke is an enriched SCLM previously modified by subduction processes.

The sloped HREE pattern of the Gornostakh dyke (Tb/Yb_n_=1.81, La/Sm_n_=1.24) indicates that partial melting likely occurred on the border of the spinel and garnet stability fields (~90 km) with a low degree of partial melting suggesting that there has not occurred lithosphere extension or delamination beneath and near the eastern margin of the Siberian craton before 1420 Ma. The younger Sette-Daban mafic sills at ca 1000 Ma^49^ were emplaced through the same SCLM because they were sampled in areas where they outcrop close to each other. Whereas those intrusions have similar primitive mantle-normalized (arc-like) profiles they have significantly higher εNd(t) values of +4.4 to +6.8 than the Gornostakh dyke and shallower spinel-bearing melting source, suggesting that their source involved a higher proportion of depleted mantle with less contribution from the enriched component due to crustal extension. The enriched LREE, LILE and isotopic compositions of both the Gornostakh and ca. 1000 Ma Sette-Daban intrusions could have been produced through interaction between asthenospheric mantle and metasomatically enriched regions within the SCLM that formed during ancient subduction events.

### Tectonic setting of the Siberia at 1420 Ma

Precambrian mafic magmatism, typically as dykes, commonly is interpreted as continental rifting and as a consequence possible break-up of the supercontinent or advocated as plume-related or both. Typically, episodes of mafic magmatism in the continental record related to extensional tectonics, but there are some examples of dykes interpreted as the development of active margin tectonics (e.g. 1.3–1.2 Ga dykes in Southwest Fennoscandian Shield^50^, and 1.39 Ga in West Australia^41^). These mafic intrusions may represent extension as a reflection of convergent and collisional tectonics and characterize by high alkalinity (presumably potassic) and island-arc trace element signatures, e.g.^51^, while continental rift-related mafic intrusions possess OIB or E-MORB trace element characteristics. At the same time, arc-like trace element profile are typical for low-Ti basalts from Phanerozoic LIPs, which commonly related to plume-lithosphere interaction (e.g.^45,52^) or to deep water and fluid recycling due to ongoing subduction^47^ – in both cases those LIPs could be a reason for continental break-up (e.g. Central Atlantic) or not (e.g. Karoo), regardless of their geodynamic causes. Thus, dyke swarms could be associated with plate-margin related processes or deep mantle geodynamics and not entirely with a continental break-up^2^.

**Figure 7:**
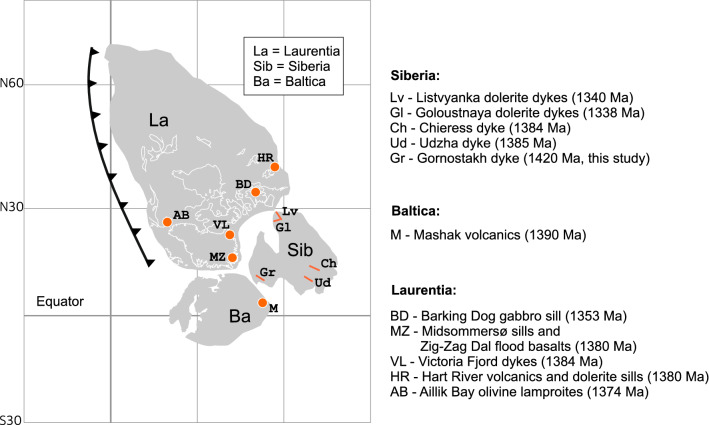
Tectonic assemblage map of core of Nuna to time if initial early Mesoproterozoic breakup events (ca. 1400) after^5,7^ with magmatic events ca 1400–1350 Ma. Ages of magmatic events from: Gl and Lv^18^, Ch^16^, Ud^17^, M^34^, AB^36^, BD^8^, MZ and VL^35^, HR^37^.

The Mesoproterozoic are considered as a period of relative tectonic quiescence in southeastern Siberia, characterized by the formation of extensive intra-cratonic basins with subsequent changing to a passive margin setting in the Neoproterosoic along the eastern margin of the craton. Paleogeographic reconstructions at ca. 1400 Ma suggest that the Laurentia, Baltica, and Siberia (Fig. 7) composed the Nuna supercontinent core^5,8,9^, though some paleoreconstructions assumes that there be a gap between Siberia and Laurentia in the Paleo- Mesoproterozoic^6^. Nuna’s lifespan is a highly debated topic in literature and there are at least two contrasting points of view. Roberts^2^ overviewed this issue and based on several pieces of evidence, such as passive margin low abundance^53^ and continental growth rate suggested that Nuna break-up had started at ca. 1.3 Ga and then fragments of Nuna reamalgamated to produce Rodinia at ca. 1.1–0.9 Ga. Others based on paleomagnetic and geological studies have argued that the interior of the supercontinent was affected by extension between 1.6 and 1.3 Ga, and finally, break-up took place around 1.3 Ga^5,54–56^. In any case, most of the authors pointed the similarity between Nuna and Rodinia configuration and have suggested a lack of large-scale movement during the supercontinental fragmentation and assembly^5^.

Towards the Siberia margin, there are few dykes recognized in the age interval 1.42 to 1.35 Ga including studied Gornostakh dyke, Chieress, Udzha, and Listvyanka dykes, and on the adjacent to Siberia – Laurentia and Baltica (Fig. 7). Since according to paleogeographical reconstructions Siberia was juxtaposed to Laurentia by the south margin and to Baltica by the southeastern margin, the active margin tectonic setting for mafic magmatism is considered unlikely. Thus, the arc-like chemical composition of the Gornostakh dyke, is considered as a feature of LIP, and flood basalt province could be proposed at ca. 1.4 Ga on the southeastern Siberian craton. Assuming Siberia – Baltica connection Gornostakh magmatism could be associated with 1.39 Ga Mashak LIP in the southeast of Baltica^34^. Siberia – Laurentia connection is attributable mainly to close in age igneous provinces from Greenland (Zig-Zag Dal and Midsommersø sills and flood basalts^35^), western Laurentia (Hart River sills^37^), and Aillik Bay olivine lamproites^36^. The widespread magmatism in Laurentia-Siberia-Baltica (and adjacent cratons) in the period of 1.42 -- 1.35 Ga is also advocated by the presence of low-Ti basalts with negative Nb anomalies among the dykes and sills mentioned above (Udzha paleorift, Hart River sills and Midsommersø sills), some of which have strong subduction trace elements signatures (e.g. Aillik Bay lamproites) that were ascribed to storage in the peri-cratonic metasomatized mantle lithosphere as a vestige of a Paleoproterozoic collisional event^36^. At the same time, such widespread intracontinental magmatic activity could be initiated by extensional tectonic, which conforms to several paleo-reconstructions^5,34,37^. The western Laurentia magmatic event dated at 1382 Ma from the study of the Hart River sills is interpreted as a manifestation of rifting of Australia from Laurentia^37^. In southeastern Siberia, the magmatism is supposed slightly earlier from Gornostakh dyke on Siberia (1420 Ma) and Mashak igneous province on Baltica (1386-1390 Ma)^34^. The last one could be interpreted as beginning extension with futher rifting, which could be supported by the Mesoproterosoic extension deformation event on Sette-Daban reflected in structural unconformity between Uchur and Aimchan groups. From the other side, the link between Baltica and Laurentia (including Siberia) up to about 1270 Ma is one of the best established paleomagnetic constraints^57^ therefore Sette-Daban extensional tectonics could be related to the failed breakup between Siberia and Baltica at 1420 Ma. To futher testing this hypothesis and determine the timing of the rifting episodes in eastern Siberia and reassembling Nuna to Rodinia further geochemical, isotopic, and paleomagnetic data across the spatial range of dykes within supercontinent remains to be investigated.

## Conclusions

A unique mafic dyke discovered ca. 1419 Ma, here named the Gornostakh dyke has been identified in the southeastern margin of Siberian Craton using in situ LA-ICPMS U-Pb dating of apatite.

Current models for the Siberian Craton infer a magmatic and tectonically quiescent period between ca. 1.88 Ga and 1.3 Ga but indirect evidence from the other margins of the craton supports beginning intraplate magmatic activity which could be related to the transition from Nuna to Rodinia configuration. Obtained U-Pb age of the Gornostakh dyke is complementary to Chieress and Udzha dykes from north Siberia suggesting that the 1400-1380 Ma LIP is widespread in the Siberia craton. The minimum sedimentation age of the Uchur Group is constrained as Lower Mesoproterozoic (1419 Ma), while the previous estimate is on the 1339 Ma. The extension deformation event in Sette-Daban, which is reflected by angular dipping of Uchur Group layers is contemporaneous with the observed magmatic event. Paleogeographic reconstructions suggest that Gornostakh magmatism together with observed deformations could reflect extensional tectonics with failed attempting Baltica-Siberia break-up at 1420 Ma.

Preliminary geochemical analysis indicates that the dyke has tholeiitic compositions with arc-like trace elements profile with a significant contribution from metasomatically enriched subcontinental lithosphere previously modified by subduction processes. Altogether with paleotectonic reconstructions which indicate a lack of active margin tectonics in the area, the geochemical data of dyke composition indicates that the low-Ti Gornostakh dyke is the remnant of the large igneous province.

## Supplementary Information

Supplementary Information.
